# Long-term risk of incident autoimmune thyroid disease after COVID-19 in patients with type 2 diabetes: a multicenter propensity score–matched cohort study

**DOI:** 10.3389/fendo.2026.1916915

**Published:** 2026-08-18

**Authors:** Kuo-Chuan Hung, Hsiu-Lan Weng, Yi-Chen Lai, I-Wen Chen

**Affiliations:** 1 Department of Anesthesiology, Chi Mei Medical Center, Tainan, Taiwan; 2 Department of Anesthesiology, E-Da Hospital, I-Shou University, Kaohsiung, Taiwan; 3 Department of Anesthesiology, Chi Mei Hospital, Liouying, Tainan, Taiwan

**Keywords:** autoimmune thyroid disease, COVID-19, Graves’ disease, Hashimoto’s thyroiditis, SARS-CoV-2, type 2 diabetes mellitus

## Abstract

**Background:**

Viral infections have been implicated in thyroid autoimmunity, and COVID-19 has emerged as a possible trigger. However, current evidence is mostly limited to case reports and short-term studies evaluating broad thyroid dysfunction rather than autoimmune-specific thyroid disease. This study aimed to evaluate whether documented SARS-CoV-2 infection is associated with a delayed and increased long-term risk of incident autoimmune thyroid disease in adults with type 2 diabetes.

**Methods:**

We used the TriNetX Global Collaborative Network to conduct a retrospective multicenter cohort study of adults with type 2 diabetes who had documented COVID-19 between January 2020 and June 2022 and matched controls without a documented SARS-CoV-2 infection. Patients with a history of thyroid disease were excluded. After 1:1 propensity score matching, a 12-month landmark design was applied. Follow-up was extended from day 365 to day 2190 after cohort entry. Incident Hashimoto’s thyroiditis and Graves’ disease were defined as primary outcomes, whereas hypothyroidism and all-cause mortality were assessed as secondary outcomes.

**Results:**

After matching, 590,346 patients were included in each cohort, with a mean age of 61.8 years. During follow-up, COVID-19 was associated with higher risks of incident Hashimoto’s thyroiditis (649 vs. 511 events; HR, 1.56; 95% CI, 1.39–1.75) and Graves’ disease (467 vs. 299 events; HR, 1.92; 95% CI, 1.66–2.23; both p<0.001). Increased risks were also observed for hypothyroidism (HR, 1.61; 95% CI, 1.57–1.65) and all-cause mortality (HR, 1.68; 95% CI, 1.65–1.71). The associations persisted across the sensitivity analyses and were consistent across sex and age subgroups. Landmark analyses using 3- and 24-month event-free intervals showed stronger associations over time for outcomes. In an exploratory analysis restricted to patients with COVID-19, pre-index vaccination was associated with a lower risk of Hashimoto’s thyroiditis but not with Graves’ disease, hypothyroidism, or all-cause mortality.

**Conclusions:**

Among adults with type 2 diabetes, documented COVID-19 was associated with an increased risk of delayed-onset autoimmune thyroid disease beyond the first post-infection year. Given the observational design and low absolute event rates, these findings do not establish causality but support targeted clinical vigilance and prospective validation using antibody-confirmed outcomes.

## 1 Introduction

Autoimmune thyroid disease is the most prevalent organ-specific autoimmune disorder globally and is a leading cause of thyroid dysfunction (1–4). The two principal forms, Hashimoto thyroiditis and Graves’ disease, affect millions of individuals and contribute substantially to long-term morbidity and healthcare utilization (5–7). Autoimmune thyroid disease may manifest as hypothyroidism or hyperthyroidism and is associated with impaired quality of life and increased cardiovascular, metabolic, and neuropsychiatric burden (8–12). As these conditions often follow a chronic or relapsing course, patients frequently require lifelong biochemical monitoring and long-term treatment (9, 13, 14). Therefore, identifying modifiable or environmental factors that trigger thyroid autoimmunity is an important public health priority.

Viral infections have long been implicated as environmental triggers of autoimmune diseases, and COVID-19 has been proposed as a potential contributor to thyroid autoimmunity (15–17). Nevertheless, the evidence remains preliminary and is mainly derived from case reports and small case series of Graves’ disease and Hashimoto thyroiditis following SARS-CoV-2 infection (15–17). Several mechanisms have been proposed to explain thyroid involvement after SARS-CoV-2 infection, including ACE2- and TMPRSS2-related viral tropism for thyroid tissue, cytokine-mediated thyroid injury, microvascular damage, and immune dysregulation (18–21). In genetically susceptible individuals, SARS-CoV-2–related inflammatory and tissue-damaging processes may facilitate the loss of immune tolerance to thyroid antigens and promote autoimmune activation, potentially through mechanisms such as immune hyperstimulation and molecular mimicry (15, 20).

Accumulating evidence suggests that COVID-19 is associated with various thyroid abnormalities after infection (22–25). Previous large electronic health record studies have reported an increased risk of new-onset thyroid disease, particularly hypothyroidism and hyperthyroidism, among COVID-19 survivors (25, 26). However, previous large electronic health record studies have primarily examined broad categories of thyroid dysfunction, such as hypothyroidism, hyperthyroidism, or unspecified thyroid disease, and the available evidence is often limited by relatively short follow-up or a lack of autoimmune thyroid disease–specific outcomes (25, 26). Thus, whether COVID-19 is associated with delayed-onset autoimmune thyroid diseases, particularly Hashimoto thyroiditis and Graves’ disease, remains insufficiently characterized. Given the large population of COVID-19 survivors, clarifying this association may help identify patients who could benefit from targeted post-acute thyroiditis monitoring.

Type 2 diabetes is characterized by metabolic dysfunction, chronic low-grade inflammation, insulin resistance, and dysregulated innate and adaptive immune responses (27–29). Thyroid disorders are also more common in patients with type 2 diabetes and may adversely interact with glycemic control and cardiometabolic risk (30, 31). These biological and clinical characteristics make patients with type 2 diabetes a relevant population in which to investigate post-infectious immune-mediated thyroid sequelae. To address this evidence gap, we conducted a large multicenter cohort study to evaluate the association between COVID-19 and subsequent autoimmune thyroid disease in adults with type 2 diabetes.

## 2 Methods

### 2.1 Study design and data source

The study protocol was approved by the Institutional Review Board of Chi Mei Medical Center. The requirement for informed consent was waived because all the data were de-identified. This study was conducted in accordance with the principles of the Declaration of Helsinki. This retrospective multicenter cohort study used the TriNetX Global Collaborative Network, a federated electronic health record platform that provides de-identified patient data contributed by participating healthcare organizations across multiple countries; therefore, the study did not involve a single index hospital or a uniform COVID-19 vaccine type. The TriNetX platform has been widely used as a data source in numerous peer-reviewed studies across diverse clinical disciplines (32–34). The database contains comprehensive clinical information, including demographic characteristics, diagnoses, procedures, medication records, laboratory results, and healthcare utilization. In this study, data from participating institutions within the Global Collaborative Network were analyzed to investigate the association between documented COVID-19 infection and the subsequent development of thyroid-related outcomes in patients with type 2 diabetes mellitus.

### 2.2 Study population and exposure definition

Adults aged ≥ 18 years with a documented diagnosis of type 2 diabetes mellitus were eligible for inclusion in this study. The exposed cohort comprised patients with confirmed SARS-CoV-2 infection between January 1, 2020, and June 30, 2022. COVID-19 exposure was identified using either a recorded COVID-19 diagnosis code or a positive SARS-CoV-2 RNA laboratory test result in the TriNetX database. The control cohort included patients with type 2 diabetes mellitus who had no documented evidence of COVID-19 infection, defined as the absence of both a COVID-19 diagnosis code and a positive SARS-CoV-2 RNA test result during the same enrollment period.

The index date was defined as the earliest date on which a patient satisfied the eligibility criteria for cohort assignment. For patients in the COVID-19 cohort, the index date corresponded to the first documented COVID-19 diagnosis or first positive SARS-CoV-2 test result recorded during the study period. For patients in the control cohort, the index date was assigned according to the first qualifying healthcare encounter related to type 2 diabetes mellitus within the corresponding calendar period. Aligning the index periods between cohorts helped minimize the temporal bias associated with changes in healthcare utilization and pandemic-related practice patterns over time.

Patients with pre-existing thyroid disease or thyroid cancer were excluded to ensure that only incident thyroid outcomes were evaluated in this study. Additional exclusions were applied to reduce confounding factors from conditions affecting thyroid function or metabolic status, including thyroid surgery, thyroid-directed medications, severe chronic kidney disease, dialysis dependence, bariatric surgery, type 1 diabetes, gestational diabetes, selected head and neck malignancies, and systemic connective tissue disorders. The corresponding diagnostic criteria and code mappings used to operationalize each variable are listed in **Supplementary Table 1**.

### 2.3 Landmark design and follow-up

A 12-month landmark design was used to reduce reverse causation, early outcome misclassification, and short-term surveillance effects after acute COVID-19 infection. This design also allowed the analysis to focus specifically on delayed-onset autoimmune thyroid disease rather than acute or early post-infectious thyroid abnormalities. Patients with relevant thyroid outcomes or death during the landmark period were excluded from the primary time-to-event analysis when applicable, and therefore thyroid outcomes occurring within the first 12 months after the index date were intentionally not included in the primary analysis. Participants were observed from day 365 through day 2190 following the index date, representing a maximum of six years of follow-up after cohort entry. Outcomes occurring during this delayed follow-up window were considered incident events. Patients were followed until the first occurrence of the outcome of interest, death when applicable, the last available record, or the end of the follow-up window.

### 2.4 Propensity score matching

To reduce baseline differences between patients with and without COVID-19, propensity score matching was performed at a 1:1 ratio using a greedy nearest-neighbor algorithm with a caliper of 0.1 pooled standard deviations of the propensity score logit. All baseline covariates used for propensity score matching, including medication use and COVID-19 vaccination, were assessed during the 5-year period before the index date and were required to precede the index event. The baseline covariates used for matching included demographic characteristics, race, body mass index, comorbidities, diabetes-related complications, cardiovascular and renal conditions, respiratory disease, liver disease, malignancy, smoking-related diagnoses, alcohol-related disorders, malnutrition, medication use, COVID-19 vaccination status, laboratory markers, and thyroid-related testing or procedures. All covariates entered into the matching procedure are enumerated in **Supplementary Table 2**. Covariate balance before and after matching was assessed using standardized mean difference. A standardized mean difference of <0.10 indicated an acceptable balance. Propensity score density plots were used to visually assess the overlap between the COVID-19 and control cohorts before and after matching.

### 2.5 Outcomes

The primary outcomes were incident autoimmune thyroid disorders, including Hashimoto’s thyroiditis and Graves’ disease, that occurred during the late follow-up window. The secondary outcomes included hypothyroidism and all-cause mortality. A benign skin tumor outcome was included as a negative control outcome, and any healthcare visit during follow-up was assessed as a general healthcare utilization marker.

### 2.6 Sensitivity, subgroup, and additional analyses

To examine the stability and reliability of the main results, a series of sensitivity analyses were performed. In Model I, the analysis was limited to individuals who remained alive throughout the follow-up period to determine whether differences in mortality affected the observed associations. Model II was restricted to participants with one or more healthcare contacts during the follow-up period, thereby assessing whether the associations persisted among patients with type 2 diabetes who had documented opportunities for outcome ascertainment rather than adjusting for healthcare utilization as a covariate. Model III was confined to patients who underwent thyroid-related testing during follow-up to minimize the differences in outcome ascertainment associated with thyroid evaluation practices. Model IV restricted the control group to individuals with no documented SARS-CoV-2 infection at any point during follow-up, thereby minimizing exposure misclassification due to subsequent COVID-19 infections among controls. In addition to the 12-month landmark used in the primary analysis, landmark sensitivity analyses were performed using 3-month and 24-month landmarks to evaluate whether the estimated associations were sensitive to the length of the event-free period after the index date. Subgroup analyses were specified *a priori* and stratified by sex and age category (18–65 versus >65 years).

In an additional exploratory analysis restricted to patients with COVID-19, patients with and without documented COVID-19 vaccination before the COVID-19 index date were matched at a 1:1 ratio, and their subsequent risks of thyroid-related outcomes and all-cause mortality were compared. Vaccination recorded after the COVID-19 index date was not used to define exposure status because the temporal sequence between post-index vaccination and thyroid outcomes could not be reliably established.

### 2.7 Severity-stratified analysis and descriptive competing-risk analysis

To examine whether the observed associations were also present among patients with severe COVID-19, we conducted a severity-stratified analysis focusing on patients with severe COVID-19 and compared them with matched control participants. Severe COVID-19 was defined as an infection requiring hospitalization. The analysis used the same outcome definitions, propensity score matching strategy, and statistical procedures as those applied in the primary analyses.

Because death may preclude the subsequent diagnosis or recording of Hashimoto’s thyroiditis and Graves’ disease, we performed a descriptive competing-risk analysis for these two outcomes in the unmatched cohort using the Aalen–Johansen method, with death treated as a competing event. This analysis was conducted independently of the propensity score–matched primary analysis and was considered supportive and descriptive rather than a primary inferential approach.

### 2.8 Statistical analysis

Baseline demographic and clinical characteristics were summarized as frequencies and percentages for categorical variables and as means with standard deviations for continuous variables. In the matched cohorts, Cox proportional hazards models were fitted to the time-to-event data, with effect estimates expressed as hazard ratios (HRs) accompanied by 95% confidence intervals (CIs). The proportional hazards assumption was assessed using the built-in proportionality test available on the TriNetX platform.

For the two primary outcomes, E-values were calculated to quantify the minimum strength of association that an unmeasured confounder would need to have with both the exposure and outcome, beyond the measured covariates, to fully explain the observed associations. Higher E-values indicate greater robustness of the findings to potential unmeasured confounding.

Missing data were not imputed, and all analyses were performed using the available data within the TriNetX platform. Statistical significance for the primary outcomes was determined using two-sided p-values, with p <0.05 considered statistically significant. Because the secondary outcome, sensitivity, subgroup, landmark, exposure-gradient, pre-index vaccination, healthcare utilization, and competing risk analyses were regarded as exploratory, no correction for multiple testing was applied.

## 3 Results

### 3.1 Patient selection and baseline characteristics

**Figure 1:**
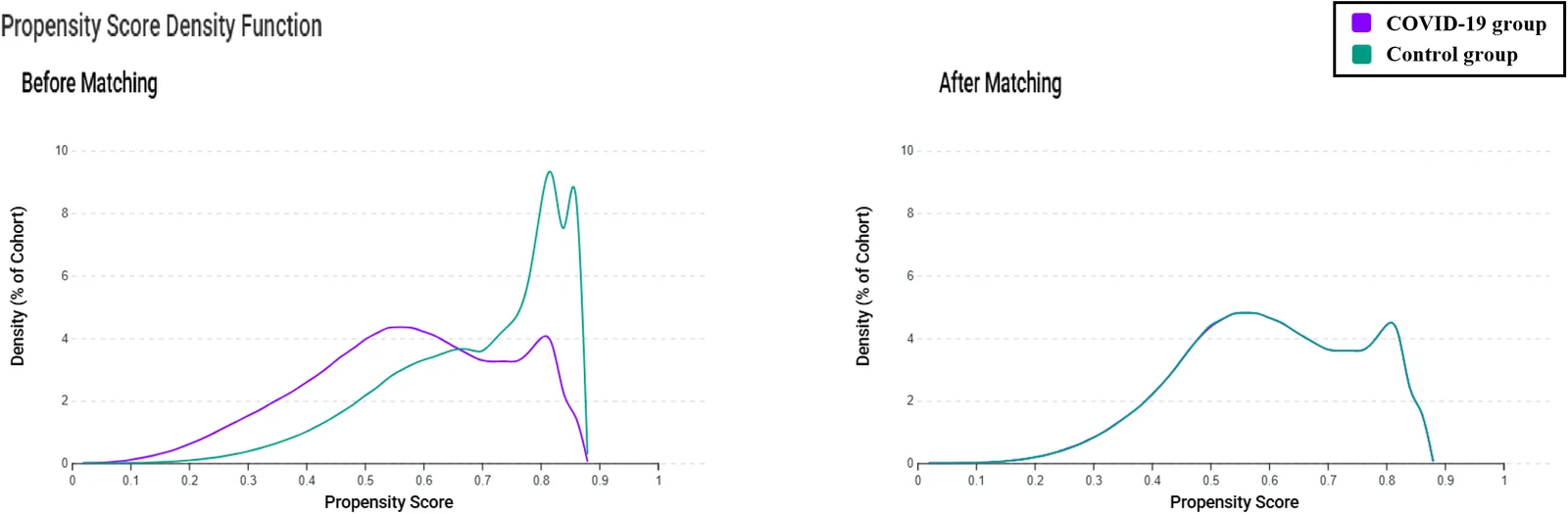
Propensity score distributions before and after matching. Propensity score density plots comparing adults with type 2 diabetes with documented COVID-19 and matched controls without documented SARS-CoV-2 infection before and after 1:1 propensity score matching. Before matching, the two cohorts showed partial separation in propensity score distributions. After matching, the distributions demonstrated substantial overlap, indicating improved covariate balance between groups.

Prior to matching, the COVID-19 cohort comprised 687,182 patients with type 2 diabetes mellitus, compared with 1,200,575 patients in the control cohort (**Table 1**). Patients with COVID-19 had a higher baseline prevalence of obesity, hypertension, dyslipidemia, ischemic heart disease, chronic kidney disease, heart failure, chronic obstructive pulmonary disease, glucocorticoid use, insulin use, and thyroid-related testing. After 1:1 propensity score matching, 590,346 patients were included in both cohorts. Baseline covariates were well balanced after matching, with all standardized mean differences being below 0.10. The mean age was 61.8 years in both groups, and women accounted for 44.4% of the COVID-19 group and 44.0% of the control group. Thyroid-related testing and head and neck ultrasound procedures were also balanced after matching the propensity scores. The propensity score distribution plots also demonstrated substantial post-matching overlap between the COVID-19 and control cohorts, further supporting adequate covariate balance (**Figure 1**).

**Table 1:** Baseline characteristics of patients with type 2 diabetes with and without COVID-19 before and after propensity score matching.

Variables	Before matching			After matching			
	COVID-19 group (n =687,182)	Control group (n = 1,200,575)	SMD		COVID-19 group (n = 590,346)	Control group (n = 590,346)	SMD
Patient characteristics							
Age at index (years)	62.0±14.2	62.3±13.8	0.019		61.8±14.4	61.8±13.7	0.001
Female	305228 (44.4)	530779 (44.2)	0.004		262197 (44.4)	259581 (44.0)	0.009
BMI>30 kg/m^2^	344604 (50.1)	398096 (33.2)	0.350		276296 (46.8)	284199 (48.1)	0.027
White	401688 (58.5)	584253 (48.7)	0.197		336053 (56.9)	344472 (58.4)	0.029
Black or African American	142356 (20.7)	195440 (16.3)	0.114		118006 (20.0)	120161 (20.4)	0.009
Asian	35511 (5.2)	87364 (7.3)	0.087		33445 (5.7)	35395 (6.0)	0.014
Comorbidities and health services							
Essential (primary) hypertension	458054 (66.7)	564815 (47.0)	0.404		371234 (62.9)	377178 (63.9)	0.021
Disorders of lipoprotein metabolism and other lipidemias	398045 (57.9)	505462 (42.1)	0.320		322358 (54.6)	326222 (55.3)	0.013
Overweight and obesity	232991 (33.9)	229407 (19.1)	0.340		176314 (29.9)	176351 (29.9)	0.000
Neoplasms	183122 (26.6)	202977 (16.9)	0.238		140292 (23.8)	141090 (23.9)	0.003
Ischemic heart diseases	158268 (23.0)	161738 (13.5)	0.249		114445 (19.4)	113312 (19.2)	0.005
Nicotine dependence	106568 (15.5)	92127 (7.7)	0.247		74950 (12.7)	73957 (12.5)	0.005
Chronic kidney disease (CKD)	83157 (12.1)	80583 (6.7)	0.185		59272 (10.0)	58532 (9.9)	0.004
Diseases of liver	81455 (11.9)	79755 (6.6)	0.181		58301 (9.9)	57137 (9.7)	0.007
Type 2 diabetes mellitus with neurological complications	82088 (11.9)	73812 (6.1)	0.203		57257 (9.7)	56558 (9.6)	0.004
Type 2 diabetes mellitus with kidney complications	75283 (11.0)	78181 (6.5)	0.158		54632 (9.3)	54060 (9.2)	0.003
Cerebrovascular diseases	74669 (10.9)	75422 (6.3)	0.164		53227 (9.0)	52569 (8.9)	0.004
Heart failure	76730 (11.2)	55141 (4.6)	0.246		47492 (8.0)	45385 (7.7)	0.013
Other chronic obstructive pulmonary disease	70361 (10.2)	52169 (4.3)	0.228		43793 (7.4)	42152 (7.1)	0.011
Type 2 diabetes mellitus with ophthalmic complications	33592 (4.9)	42076 (3.5)	0.069		25866 (4.4)	26029 (4.4)	0.001
Type 2 diabetes mellitus with circulatory complications	38486 (5.6)	32329 (2.7)	0.146		25763 (4.4)	25446 (4.3)	0.003
Noninfective enteritis and colitis	38593 (5.6)	30919 (2.6)	0.154		25679 (4.4)	24792 (4.2)	0.007
Alcohol related disorders	30661 (4.5)	21187 (1.8)	0.156		19004 (3.2)	18186 (3.1)	0.008
Malnutrition	11873 (1.7)	5306 (0.4)	0.124		5459 (0.9)	5010 (0.8)	0.008
Medications							
Antilipemic agents	350639 (51.0)	418313 (34.8)	0.331		279019 (47.3)	282363 (47.8)	0.011
Glucocorticoids	314815 (45.8)	304974 (25.4)	0.436		238092 (40.3)	237802 (40.3)	0.001
Metformin	287634 (41.9)	371116 (30.9)	0.229		236834 (40.1)	242273 (41.0)	0.019
Insulins and analogues	221678 (32.3)	204257 (17.0)	0.359		163016 (27.6)	161018 (27.3)	0.008
GLP-1 analogues	62384 (9.1)	59650 (5.0)	0.161		45974 (7.8)	45618 (7.7)	0.002
SGLT2 inhibitors	58271 (8.5)	65769 (5.5)	0.118		44753 (7.6)	45108 (7.6)	0.002
SARS-cov-2 (COVID-19) vaccine	78038 (11.4)	54827 (4.6)	0.253		43928 (7.4)	40151 (6.8)	0.025
Laboratory data							
eGFR≥60 mL/min/1.73 m²	511929 (74.5)	628695 (52.4)	0.472		422027 (71.5)	425733 (72.1)	0.014
Hemoglobin ≥12 g/dL	493129 (71.8)	547299 (45.6)	0.551		400145 (67.8)	403313 (68.3)	0.012
Albumin ≥3.5 g/dL	467450 (68.0)	503895 (42.0)	0.543		375813 (63.7)	377112 (63.9)	0.005
HbA1c≥9%	122904 (17.9)	146575 (12.2)	0.159		98761 (16.7)	99075 (16.8)	0.001
TSH 0.4–4 m[IU]/L	272828 (39.7)	289903 (24.1)	0.338		214059 (36.3)	214356 (36.3)	0.001
Thyroid-related Procedure							
TSH	166107 (24.2)	144066 (12.0)	0.320		119571 (20.3)	116792 (19.8)	0.012
Diagnostic Ultrasound Procedures of the Head and Neck	11130 (1.6)	11751 (1.0)	0.057		8316 (1.4)	8541 (1.4)	0.003

Data are presented as number (%) or mean ± standard deviation. Propensity score matching was performed at a 1:1 ratio using a greedy nearest-neighbor algorithm with a caliper of 0.1 pooled standard deviation of the propensity score logit. Standardized mean differences <0.10 indicated an acceptable covariate balance. BMI, body mass index; CKD, chronic kidney disease; COVID-19, coronavirus disease 2019; eGFR, estimated glomerular filtration rate; GLP-1, glucagon-like peptide-1; HbA1c, hemoglobin A1c; SGLT2, sodium-glucose cotransporter 2; SMD, standardized mean difference; TSH, thyroid-stimulating hormone.

### 3.2 Primary and secondary outcomes

During late follow-up, Hashimoto’s thyroiditis occurred in 649 COVID-19 patients and 511 controls (0.11% vs 0.09%) (**Table 2**). COVID-19 was associated with a higher risk of incident Hashimoto’s thyroiditis than no documented SARS-CoV-2 infection (HR, 1.56; 95% CI, 1.39–1.75; p<0.001). The E-values were 2.58 for the point estimate and 2.04 for the lower confidence limit, suggesting that only an unmeasured confounder strongly associated with both COVID-19 and Hashimoto’s thyroiditis could fully account for this association beyond the measured covariates. Similarly, Graves’ disease occurred in 467 COVID-19 patients and 299 controls (0.08% vs 0.05%). Risk was nearly doubled (HR 1.92; 95% CI 1.66–2.23; p<0.001), with E-values of 3.39 (lower CI 2.58), indicating moderate robustness.

**Table 2:** Association between COVID-19 and long-term risks of incident autoimmune thyroid disease and related outcomes.

Outcome	COVID-19 group (n=590,346)	Control group (n=590,346)	HR (95% CI)	*p* value
	Events (%)	Events (%)		
Primary and secondary outcomes				
Hashimoto’s thyroiditis	649 (0.11)	511 (0.09)	1.56 (1.39–1.75)	<0.001
Graves’ disease	467 (0.08)	299 (0.05)	1.92 (1.66–2.23)	<0.001
Hypothyroidism	15,356 (2.60)	11,612 (1.97)	1.61 (1.57–1.65)	<0.001
Mortality	38,876 (6.59)	28,234 (4.78)	1.68 (1.65–1.71)	<0.001
Negative control outcome and healthcare-utilization marker				
Benign skin tumor	7,823 (1.33)	8,019 (1.36)	1.14 (1.10–1.17)	<0.001
Any healthcare visit	524,026 (88.77)	530,427 (89.85)	0.99 (0.99–1.00)	<0.001

Data are presented as number of events (%). Benign skin tumors were included as a negative control outcome, and any healthcare visit was assessed as a general healthcare utilization marker. CI, confidence interval; COVID-19, coronavirus disease 2019; HR, hazard ratio.

Additional analyses demonstrated that COVID-19 was linked to an increased risk of hypothyroidism (HR, 1.61; p<0.001) and all-cause mortality (HR, 1.68; p<0.001). The negative control outcome, benign skin tumors, was associated with a modest increase in risk among patients with COVID-19 (HR, 1.14; p<0.001). In contrast, although a statistically significant difference was observed in overall healthcare utilization, the effect size was negligible (HR, 0.99; p<0.001), suggesting that healthcare utilization was broadly comparable between the two cohorts during follow-up.

### 3.3 Sensitivity analyses

The associations between COVID-19 and incident thyroid outcomes remained consistent across the sensitivity analyses (**Table 3**). In the survivor-only analysis, COVID-19 remained associated with Hashimoto’s thyroiditis (HR, 1.47; p<0.001) and Graves’ disease (HR, 1.91; p<0.001). Similar results were observed among patients with at least one healthcare visit during the follow-up for Hashimoto’s thyroiditis (HR, 1.44; p<0.001) and Graves’ disease (HR, 1.89; p<0.001). Among patients who underwent thyroid-related testing during follow-up, the associations persisted for Hashimoto’s thyroiditis (HR, 1.53; p<0.001) and Graves’ disease (HR, 1.82; p<0.001). Notably, when the control cohort was restricted to patients without documented SARS-CoV-2 infection at any point during follow-up, the associations became even stronger, with elevated risks observed for Hashimoto’s thyroiditis (HR, 1.68; p<0.001) and particularly for Graves’ disease (HR, 2.25; p<0.001), further reinforcing the robustness of the primary findings and minimizing the potential influence of undetected SARS-CoV-2 exposure in the control group.

**Table 3:** Sensitivity analyses for the association between COVID-19 and long-term risks of incident autoimmune thyroid disease and related outcomes.

Outcomes	Model I (n=554,620)		Model II (n=521,073)		Model III (n=233,617)		Model IV (n=501,849)	
	HR (95% CI)	*p* value	HR (95% CI)	*p* value	HR (95% CI)	*p* value	HR (95% CI)	*p* value
Hashimoto’s thyroiditis	1.47 (1.31–1.66)	<0.001	1.44 (1.28–1.62)	<0.001	1.53 (1.35–1.73)	<0.001	1.68 (1.47–1.91)	<0.001
Graves’ disease	1.91 (1.64–2.22)	<0.001	1.89 (1.63–2.19)	<0.001	1.82 (1.55–2.13)	<0.001	2.25 (1.90–2.66)	<0.001
Hypothyroidism	1.61 (1.57–1.65)	<0.001	1.61 (1.57–1.65)	<0.001	1.59 (1.54–1.63)	<0.001	1.93 (1.87–1.98)	<0.001
Mortality	NA	NA	1.72 (1.69–1.74)	<0.001	1.75 (1.71–1.80)	<0.001	1.93 (1.90–1.97)	<0.001
Benign skin tumor	1.14 (1.10–1.18)	<0.001	1.12 (1.08–1.15)	<0.001	1.15 (1.10–1.19)	<0.001	1.17 (1.13–1.22)	<0.001
Any healthcare visit	0.99 (0.98–0.99)	<0.001	0.99 (0.98–0.99)	<0.001	1.02 (1.01–1.03)	<0.001	1.02 (1.01–1.02)	<0.001

Model I included only patients who survived during follow-up. Model II included only patients with at least one healthcare visit during the follow-up period. Model III was restricted to patients who underwent thyroid-related tests during follow-up. Model IV restricted the control cohort to individuals with no documented SARS-CoV-2 infection at any point during follow-up to reduce exposure to misclassification. CI, confidence interval; COVID-19, coronavirus disease 2019; HR, hazard ratio; NA, not applicable.

Landmark analyses using 3-month and 24-month landmarks yielded consistent findings and demonstrated a progressive increase in risk over time (**Table 4**). For Hashimoto’s thyroiditis, the HRs were 1.54 and 1.77, respectively (all p<0.001). For Graves’ disease, the corresponding hazard ratios were 1.69 and 2.25, respectively (all p<0.001). A similar stepwise increase was observed across the primary and secondary thyroid outcomes, whereas the estimates for the negative control outcome and healthcare utilization marker remained largely unchanged across landmark periods.

**Table 4:** Landmark analyses of the association between COVID-19 and long-term thyroid outcomes.

Outcomes	3-m		12-m‡		24-m	
	HR (95% CI)	*p* value	HR (95% CI)	*p* value	HR (95% CI)	*p* value
Hashimoto’s thyroiditis	1.54 (1.39–1.71)	<0.001	1.56 (1.39–1.75)	<0.001	1.77 (1.51–2.08)	<0.001
Graves’ disease	1.69 (1.48–1.93)	<0.001	1.92 (1.66–2.23)	<0.001	2.25 (1.86–2.73)	<0.001
Hypothyroidism	1.56 (1.53–1.60)	<0.001	1.61 (1.57–1.65)	<0.001	1.84 (1.78–1.90)	<0.001
Mortality	1.53 (1.51–1.55)	<0.001	1.68 (1.65–1.71)	<0.001	1.92 (1.88–1.95)	<0.001
Benign skin tumor	1.12 (1.09–1.16)	<0.001	1.14 (1.10–1.17)	<0.001	1.12 (1.09–1.16)	<0.001
Any healthcare visit	1.00 (1.00–1.01)	0.043	0.99 (0.99–1.00)	<0.001	1.00 (1.00–1.01)	0.239

Landmark analyses were performed using 3- and 24-month event-free periods after the index dates. ‡The 12-month landmark analysis was the primary analysis. CI, confidence interval; COVID-19, coronavirus disease 2019; HR, hazard ratio.

### 3.4 Subgroup analyses

In sex-stratified analyses (**Table 5**), the association between COVID-19 and Hashimoto’s thyroiditis was observed in both male (HR, 1.53; p<0.001) and female (HR, 1.63; p < 0.001) patients, without evidence of significant interaction. Similar findings were observed for Graves’ disease in male (HR, 1.92; p<0.001) and female (HR, 1.86; p < 0.001) patients. In age-stratified analyses (**Table 6**), COVID-19 was associated with Hashimoto’s thyroiditis among patients aged 18–65 years (HR, 1.52; p<0.001) and those aged >65 years (HR, 1.66; p<0.001), with no significant interaction (p >0.05). The association with Graves’ disease was also consistent in both age groups (18–65 years: HR, 1.86; p<0.001; >65 years: HR, 1.88; p<0.001), without evidence of significant interaction (p for interaction >0.05).

**Table 5:** Sex-stratified analyses of the association between COVID-19 and long-term risks of incident autoimmune thyroid disease and related outcomes.

Outcomes	Male (n=330,032 for each group)		Female (n=239,600 for each group)		P for interaction
	HR (95% CI)	*p* value	HR (95% CI)	*p* value	
Hashimoto’s thyroiditis	1.53 (1.25–1.88)	<0.001	1.63 (1.40–1.90)	<0.001	0.626
Graves’ disease	1.92 (1.49–2.49)	<0.001	1.86 (1.54–2.24)	<0.001	0.847
Hypothyroidism	1.65 (1.60–1.71)	<0.001	1.59 (1.53–1.65)	<0.001	0.149
Mortality	1.66 (1.63–1.70)	<0.001	1.65 (1.61–1.70)	<0.001	0.731
Benign skin tumor	1.09 (1.05–1.14)	<0.001	1.22 (1.16–1.28)	<0.001	<0.001
Any healthcare visit	0.99 (0.98–0.99)	<0.001	1.01 (1.00–1.02)	<0.001	<0.001

Data are presented as hazard ratios with 95% confidence intervals and p-values. CI, confidence interval; COVID-19, coronavirus disease 2019; HR, hazard ratio.

**Table 6:** Age-stratified analyses of the association between COVID-19 and long-term risks of incident autoimmune thyroid disease and related outcomes.

Outcomes	18-65 (n=248,613 for each group)		>65 (n=338,400 for each group)		P for interaction
	HR (95% CI)	*p* value	HR (95% CI)	*p* value	
Hashimoto’s thyroiditis	1.52 (1.29–1.80)	<0.001	1.66 (1.39–1.97)	<0.001	0.477
Graves’ disease	1.86 (1.51–2.30)	<0.001	1.88 (1.52–2.32)	<0.001	0.944
Hypothyroidism	1.61 (1.54–1.68)	<0.001	1.62 (1.57–1.67)	<0.001	0.820
Mortality	1.75 (1.68–1.82)	<0.001	1.63 (1.60–1.66)	<0.001	0.002
Benign skin tumor	1.21 (1.15–1.28)	<0.001	1.11 (1.07–1.16)	<0.001	0.013
Any healthcare visit	1.04 (1.03–1.05)	<0.001	0.96 (0.96–0.97)	<0.001	<0.001

Data are presented as hazard ratios with 95% confidence intervals and p-values. CI, confidence interval; COVID-19, coronavirus disease 2019; HR, hazard ratio.

### 3.5 Severity-stratified analysis and descriptive competing-risk analysis

In the severity-stratified analysis (**Table 7**), severe COVID-19 was associated with higher risks of Hashimoto’s thyroiditis (HR, 1.60; p<0.001), Graves’ disease (HR, 1.99; p<0.001), hypothyroidism (HR, 1.66; p<0.001), and all-cause mortality (HR, 2.02; p<0.001). However, these estimates were only modestly higher than those in the primary analysis and should not be interpreted as evidence of a clear severity-dependent gradient. Estimates for the negative control outcome and healthcare utilization remained close to null (benign skin tumor: HR, 1.15; p<0.001; any healthcare visit: HR, 1.01; p=0.024), suggesting minimal differences by COVID-19 severity.

**Table 7:** Exposure-gradient analysis comparing patients with severe COVID-19 and matched controls.

Outcome	Severe COVID-19 group (n=312,012)	Control group (n=312,012)	HR (95% CI)	*p* value	*Primary analysis‡*
	Events (%)	Events (%)			
Hashimoto’s thyroiditis	313 (0.10)	253 (0.08)	1.60 (1.35–1.89)	<0.001	1.56 (1.39–1.75)
Graves’ disease	220 (0.07)	145 (0.05)	1.99 (1.60–2.46)	<0.001	1.92 (1.66–2.23)
Hypothyroidism	8,262 (2.65)	6,339 (2.03)	1.66 (1.61–1.72)	<0.001	1.61 (1.57–1.65)
Mortality	28,923 (9.27)	18,194 (5.83)	2.02 (1.98–2.06)	<0.001	1.68 (1.65–1.71)
Benign skin tumor	3,963 (1.27)	4,172 (1.34)	1.15 (1.10–1.20)	<0.001	1.14 (1.10–1.17)
Any healthcare visit	270,118 (86.57)	278,723 (89.33)	1.01 (1.00–1.01)	0.024	0.99 (0.99–1.00)

Data are presented as number of events (%). Severe COVID-19 was defined as COVID-19 requiring hospitalization. *‡*The primary-analysis estimates are shown for comparison with the overall COVID-19 cohort. CI, confidence interval; COVID-19, coronavirus disease 2019; HR, hazard ratio.

**Figure 2:**
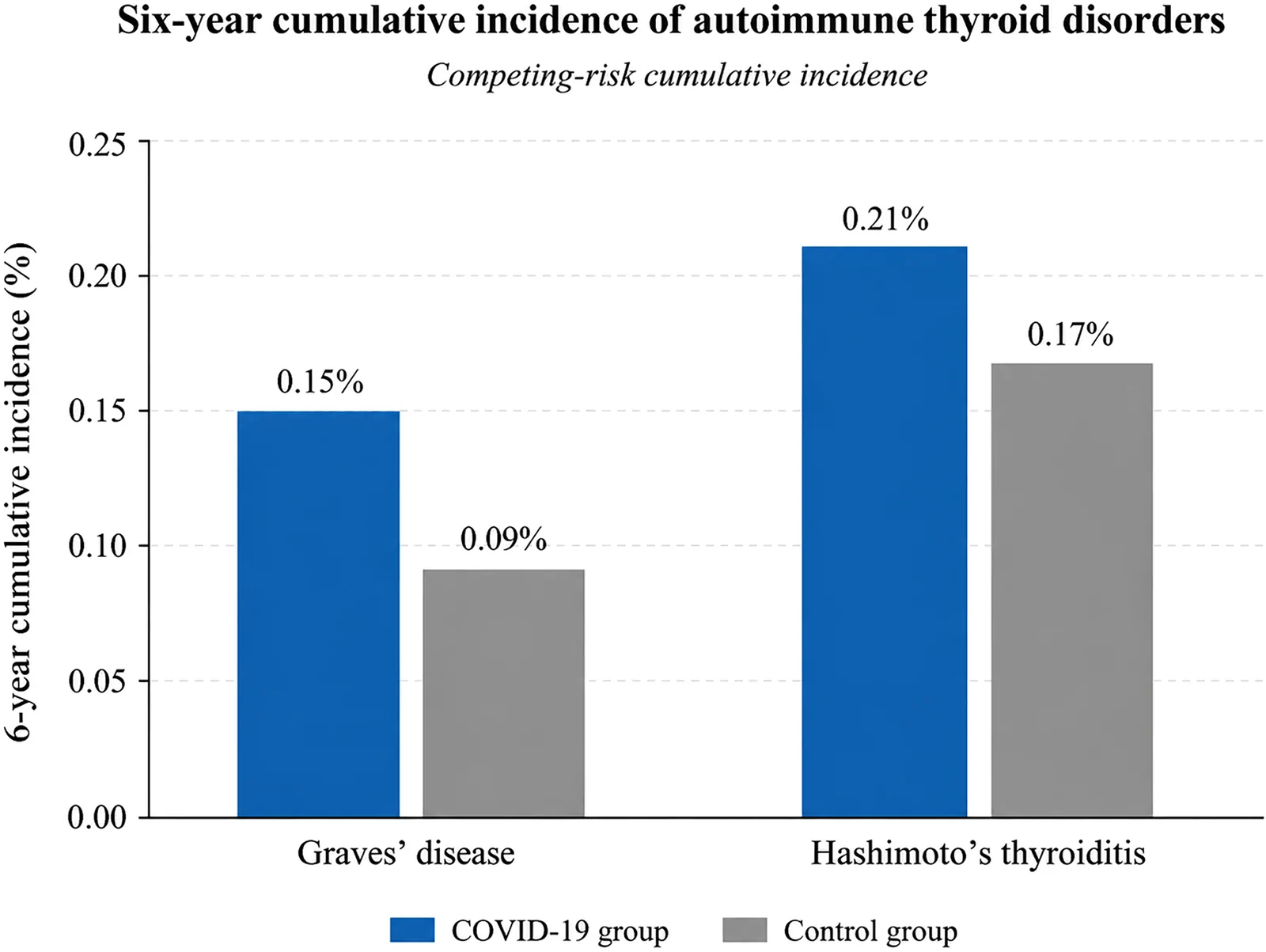
Descriptive competing-risk analysis for incident autoimmune thyroid disease with death as a competing event. Aalen–Johansen cumulative incidence analysis showing the long-term incidence of Graves’ disease and Hashimoto’s thyroiditis among adults with type 2 diabetes with and without COVID-19 in an unmatched cohort. Death was treated as a competing event. Follow-up began 365 days after the index date and continued through day 2190. This analysis was conducted independently in the unmatched cohort and is presented for descriptive purposes only; formal comparisons between groups were not performed.

When death was modeled as a competing event in the descriptive competing-risk analysis, the 6-year cumulative incidence of autoimmune thyroid disorders remained higher in the COVID-19 group than that in the control group. The cumulative incidence of Graves’ disease was 0.15% in the COVID-19 group compared with 0.09% in the control group (**Figure 2**). For Hashimoto thyroiditis, the corresponding cumulative incidences were 0.21% and 0.17%, respectively. Although the absolute differences were small, the direction of the competing risk estimates was consistent with the primary Cox regression analyses, supporting a higher long-term burden of incident autoimmune thyroid disorders among patients with type 2 diabetes after COVID-19.

### 3.6 Pre-index COVID-19 vaccination analysis

In the exploratory analysis restricted to patients with COVID-19, 108,378 patients with documented pre-index COVID-19 vaccination were matched to an equal number of patients without documented pre-index vaccination (**Table 8**). Pre-index vaccination was associated with a lower risk of Hashimoto’s thyroiditis (96 vs. 117 events; HR, 0.70; p=0.010). No significant associations were observed for Graves’ disease (HR, 0.96; p=0.790), hypothyroidism (HR, 1.02; p=0.466), or all-cause mortality (HR, 0.98; p=0.160). The pre-index vaccinated group had higher risks of the negative control outcome of benign skin tumor (HR, 1.21; p<0.001) and any healthcare visit (HR, 1.33; p<0.001).

**Table 8:** Association of pre-index COVID-19 vaccination with risks of incident thyroid disorders among patients with COVID-19.

Outcome	Pre-index vaccinated group (n = 108,378)	Pre-index unvaccinated group (n = 108,378)	HR (95% CI)	*p* value
	Events (%)	Events (%)		
Hashimoto’s thyroiditis	96 (0.09)	117 (0.11)	0.70 (0.54–0.92)	0.010
Graves’ disease	92 (0.09)	83 (0.08)	0.96 (0.71–1.29)	0.790
Hypothyroidism	3,436 (3.17)	2,935 (2.71)	1.02 (0.97–1.07)	0.466
Mortality	9,360 (8.64)	8,330 (7.69)	0.98 (0.95–1.01)	0.160
Benign skin tumor	2,227 (2.06)	1,621 (1.50)	1.21 (1.13–1.29)	<0.001
Any healthcare visit	104,071 (96.03)	98,271 (90.67)	1.33 (1.32–1.34)	<0.001

COVID-19 vaccination was defined as any documented vaccination before the COVID-19 index date. The pre-index vaccinated and unvaccinated groups were matched at a 1:1 ratio using propensity score matching. CI, confidence interval; COVID-19, coronavirus disease 2019; HR, hazard ratio.

## 4 Discussion

In this large multicenter propensity score–matched cohort of adults with type 2 diabetes, prior COVID-19 was associated with a higher long-term risk of incident autoimmune thyroid disease, including Hashimoto’s thyroiditis and Graves’ disease, as well as related thyroid outcomes. This association emerged beyond the first post-infection year, strengthened in longer landmark analyses, and persisted after accounting for healthcare contact and thyroid testing results. In the exploratory COVID-19 cohort analysis, pre-index vaccination was associated with a lower risk of Hashimoto’s thyroiditis, but not with Graves’ disease, hypothyroidism, or mortality. Because vaccinated patients had greater healthcare utilization and more negative-control events, this finding should be interpreted cautiously. The healthcare-utilization marker was close to null, whereas the association with the negative control outcome was substantially weaker than those observed for autoimmune thyroid outcomes. These findings extend the evidence on post-COVID thyroid dysfunction by identifying delayed, autoimmune-specific thyroid disease in a metabolically vulnerable population.

Our results are consistent with emerging population-level evidence that SARS-CoV-2 infection may be followed by thyroid morbidity across systematic reviews, mechanistic reviews, and population-based studies (16, 17, 22–26). Recent electronic health record studies have reported a higher risk of new-onset thyroid disease and thyroid dysfunction among COVID-19 survivors, including hypothyroid and hyperthyroid phenotypes (25, 26). However, these prior studies did not specifically evaluate autoimmune thyroid disease as a distinct outcome category, leaving the long-term risks of Hashimoto’s thyroiditis and Graves’ disease insufficiently defined. Our estimates for hypothyroidism were directionally consistent with those of prior reports (25, 26), suggesting that the association between COVID-19 and subsequent thyroid morbidity is reproducible across different healthcare systems and analytical frameworks. However, the present study extends the literature in two important ways. First, rather than relying only on broad thyroid dysfunction categories, we specifically evaluated autoimmune thyroid diseases, including Hashimoto’s thyroiditis and Graves’ disease, which represent chronic immune-mediated conditions with distinct clinical implications. Second, our findings provide large-scale cohort evidence for hypotheses previously suggested mainly by case reports, small series, and reviews describing thyroid autoimmunity after COVID-19 (15–24). By combining propensity score matching with a delayed landmark design, our study extends prior work beyond early or transient thyroid abnormalities and indicates that SARS-CoV-2 infection may be linked to later autoimmune thyroid outcomes in patients with type 2 diabetes. However, this design intentionally did not capture autoimmune thyroid disease diagnosed during the first post-infection year.

The relationship between thyroid disease and COVID-19 may be bidirectional. Although SARS-CoV-2 infection may trigger subsequent thyroid dysfunction and autoimmunity, pre-existing thyroid disease, particularly hypothyroidism, has also been associated with severe COVID-19 and poorer clinical outcomes in previous meta-analyses (35, 36). These findings suggest that baseline thyroid status may influence susceptibility to acute COVID-19 and potentially its post-acute consequences. However, evidence regarding long COVID remains limited, and our study could not directly evaluate this direction of association because patients with pre-existing thyroid disease were excluded from the study cohort at baseline. Although a case series described late-onset thyroid disturbances following COVID-19 vaccination (37), its uncontrolled design precluded estimation of incidence, comparative risk, or causality. In our matched analysis, pre-index vaccination was associated with a lower risk of Hashimoto’s thyroiditis but not with Graves’ disease, hypothyroidism, or mortality; however, this exploratory finding should be interpreted cautiously.

We specifically studied patients with type 2 diabetes because metabolic dysfunction, chronic systemic inflammation, and altered immune regulation are well-recognized features of this population (27–29, 38, 39). Thyroid dysfunction is also common in patients with type 2 diabetes and may adversely interact with glycemic control and cardiometabolic risk (30, 31). Together, these characteristics provide a biologically relevant setting in which SARS-CoV-2–related immune perturbation could contribute to autoimmune thyroid disease (15–17, 20). In addition to this biological rationale, patients with type 2 diabetes often have more regular healthcare contact, repeated laboratory monitoring, and structured follow-up for metabolic and cardiovascular comorbidities, which may improve the capture of delayed thyroid outcomes. This setting may also reduce under-ascertainment of chronic endocrine sequelae that could be missed in populations with less consistent medical engagement. In addition, thyroid dysfunction may have greater clinical consequences in patients with type 2 diabetes because it can interact with glycemic control, cardiovascular risk, renal disease, and medication management (12, 30, 31). However, the restriction to this metabolically vulnerable population also limits direct extrapolation to non-diabetic individuals, in whom baseline risk, healthcare utilization patterns, and effect size may differ. Accordingly, the present findings should be interpreted as specific to adults with type 2 diabetes and require external validation before being generalized to non-diabetic populations.

Surveillance bias is an important consideration in studies evaluating the outcomes. Individuals with documented COVID-19 may undergo more frequent follow-up visits, laboratory testing, or diagnostic assessments, potentially increasing the likelihood of thyroid disease identification. However, several findings from our study suggest that surveillance bias alone is unlikely to explain the observed associations. First, overall healthcare utilization during follow-up was nearly identical between the matched COVID-19 and control groups, indicating similar opportunities for detecting disease. Second, the negative control outcome, benign skin tumors, demonstrated only a modest association and was substantially weaker than the associations observed for Hashimoto’s thyroiditis and Graves’ disease. If increased medical attention was the primary explanation, a comparable increase would be expected for conditions unrelated to COVID-19 but commonly detected during routine clinical care. Third, although thyroid-related testing was more frequent in the COVID-19 cohort before matching, thyroid-stimulating hormone testing, normal thyroid-stimulating hormone measurements, and head and neck ultrasound examinations were well balanced between the cohorts after propensity score matching. Moreover, the associations with Hashimoto’s thyroiditis and Graves’ disease persisted in the sensitivity analysis restricted to patients who underwent thyroid-related testing during follow-up. Taken together, these findings suggest that the increased risk observed was not merely a consequence of greater diagnostic intensity following COVID-19, although residual surveillance bias cannot be completely excluded.

The temporal pattern of the landmark analyses further argues against a simple ascertainment bias. In the primary analysis, follow-up started one year after the index date, excluding the acute and early post-acute periods when patients recovering from COVID-19 are most likely to undergo intensive clinical monitoring. If the observed associations were primarily driven by increased early testing, the effect estimates would weaken as the landmark interval increased. Instead, the associations for both Hashimoto’s thyroiditis and Graves’ disease became progressively stronger across the 3-, 12-, and 24-month landmark analyses, whereas the estimates for the negative control outcome and healthcare utilization marker remained relatively unchanged. This pattern is more consistent with a delayed immune-mediated mechanism than with transient diagnostic artifacts. In addition, the competing risk analysis showed that the higher cumulative incidence of autoimmune thyroid disease among individuals with COVID-19 persisted after accounting for death as a competing event, indicating that differential mortality was unlikely to explain the findings. Finally, the E-values suggested that a substantial degree of unmeasured confounding was necessary to fully account for the observed associations. Although no single analysis can completely eliminate bias, the consistency of these results strengthens the evidence for a delayed post-COVID autoimmune thyroid signal.

Although the absolute incidence of autoimmune thyroid disease is low, the widespread exposure to SARS-CoV-2 means that even modest increases in relative risk could have important public health consequences. This may be especially relevant for individuals with type 2 diabetes, who often have multiple metabolic, cardiovascular, and renal comorbidities and may be more susceptible to immune-related complications than those without diabetes (27, 29). The delayed onset observed in this study also has clinical significance because autoimmune thyroid diseases frequently require prolonged biochemical monitoring and long-term treatment (5–14). Thyroid dysfunction developing months or even years after COVID-19 should not automatically be considered unrelated to prior infection, particularly when symptoms or laboratory findings indicate a possible autoimmune origin of the disease. However, these results do not support routine thyroid screening in all patients after COVID-19. Given the low absolute event rates, widespread screening could lead to unnecessary costs, false-positive results, and increased patient anxiety. A more appropriate strategy may be to maintain greater clinical vigilance and perform targeted thyroid assessments in symptomatic or high-risk patients with type 2 diabetes, while future prospective studies determine which individuals may benefit the most from post-COVID thyroid surveillance.

Several limitations should be considered. First, because this was an observational EHR-based study, causality cannot be inferred and residual confounding may persist despite extensive propensity score matching, sensitivity analyses, and E-value assessment. Second, thyroid outcomes were identified from diagnostic codes and available clinical records rather than through adjudicated case reviews. We could not systematically confirm Hashimoto’s thyroiditis or Graves’ disease using thyroid autoantibody titers, imaging findings, medication patterns, or clinical adjudication. Because this retrospective study relied on de-identified, routinely collected data from a federated electronic health record network and did not permit study-mandated serological testing or additional specimen collection; therefore, outcome misclassification could not be excluded. Third, although severe COVID-19 was associated with slightly higher point estimates for autoimmune thyroid outcomes than those observed in the primary analysis, no formal comparison between severe and non-severe COVID-19 was performed, and a clear severity-dependent gradient could not be established. This may indicate that autoimmune triggering is not strictly dependent on acute infection severity, but it may also reflect imperfect severity classification or limited statistical power for rare outcomes such as autoimmune diseases. Fourth, although vaccination documented before the COVID-19 index date was evaluated in an exploratory matched analysis, detailed information regarding vaccine type, number of doses, exact timing, post-infection vaccination, viral variants, reinfections, and evolving COVID-19 treatments remained incomplete, limiting causal interpretation and the assessment of effect modification across pandemic phases and calendar periods. Finally, the cohort was restricted to adults with type 2 diabetes, a metabolically and immunologically vulnerable population. Therefore, the absolute risks and relative estimates should not be directly generalized to non-diabetic populations without external validation using other datasets.

## 5 Conclusion

In conclusion, among adults with type 2 diabetes, documented COVID-19 was associated with increased long-term risks of delayed-onset Hashimoto’s thyroiditis and Graves’ disease beyond the first post-infection year. Given the observational design and low absolute event rates, these findings do not establish causality or support routine thyroid screening. Instead, they support targeted clinical vigilance in symptomatic or high-risk patients. Prospective studies using antibody-confirmed outcomes are needed to validate these observations and clarify their clinical implications.

## Data Availability

The datasets presented in this article are not readily available because the datasets generated and/or analyzed during the current study are not publicly available and cannot be shared by the corresponding author because the data were obtained from the TriNetX Global Collaborative Network under data-use restrictions. TriNetX provides access only to de-identified electronic health record data within its secure federated platform, and patient-level data cannot be downloaded, redistributed, or transferred outside the platform. Access to the underlying data is subject to TriNetX data governance policies and institutional agreements. Aggregated results and code definitions used in this study are available in the manuscript and supplementary materials. Requests to access the datasets should be directed to https://live.trinetx.com.
