# Supplementary material for: Long-term risk of incident autoimmune thyroid disease after COVID-19 in patients with type 2 diabetes: a multicenter propensity score–matched cohort study
Source: Front Endocrinol (Lausanne). 2026 Aug 18;17:1916915. doi: 10.3389/fendo.2026.1916915 (PMC13529569; doi:10.3389/fendo.2026.1916915)
Supplement: Supplementary file 1 [file Table1.docx]

**Supplemental Table 1. Codes used to define cohort eligibility, exposure, and exclusion criteria**

| **Criterion** | **Code set / definition** |
| --- | --- |
| Age ≥18 years | TriNetX demographic variable |
| Type 2 diabetes mellitus | ICD-10-CM E11 |
| COVID-19 exposure | ICD-10-CM U07.1; TriNetX laboratory term TNX:9088, SARS coronavirus 2 and related RNA [Presence] |
| Control cohort without documented COVID-19 | Absence of ICD-10-CM U07.1 and absence of TriNetX laboratory term TNX:9088 during the enrollment period |
| Baseline healthcare encounter availability | TriNetX visit term TNX |
| Pre-existing thyroid disease | ICD-10-CM E00–E07 |
| Thyroid cancer | ICD-10-CM C73 |
| Advanced chronic kidney disease | ICD-10-CM N18.4, N18.5 |
| End-stage renal disease or dialysis dependence | ICD-10-CM N18.6, Z99.2 |
| Bariatric surgery status | ICD-10-CM Z98.84 |
| Non–type 2 diabetes classification | ICD-10-CM E10, O24.4 |
| Early death or uncertain mortality coding during landmark period | TriNetX deceased status; ICD-10-CM R99 |
| Postpartum thyroiditis | ICD-10-CM O90.5 |
| Postprocedural hypothyroidism | ICD-10-CM E89.0 |
| Prior neck operation | SNOMED CT 37508003 |
| Prior thyroid resection | ICD-10-PCS 0GTK |
| Carcinoma in situ of thyroid and other endocrine glands | ICD-10-CM D09.3 |
| Benign neoplasm of thyroid gland | ICD-10-CM D34 |
| Neoplasm of uncertain behavior of thyroid gland | ICD-10-CM D44.0 |
| Prior thyroid-directed medication use | RxNorm 10582, levothyroxine; RxNorm 10814, liothyronine; RxNorm 6835, methimazole; RxNorm 8794, propylthiouracil |
| Systemic connective tissue disorders | ICD-10-CM M30–M36 |
| Malignant neoplasm of head, face, and neck | ICD-10-CM C76.0 |
| Malignant neoplasms of lip, oral cavity, and pharynx | ICD-10-CM C00–C14 |
| Malignant neoplasm of nasal cavity and middle ear | ICD-10-CM C30 |
| Malignant neoplasm of accessory sinuses | ICD-10-CM C31 |
| Malignant neoplasm of larynx | ICD-10-CM C32 |

**Supplemental Table 2. Codes for variables used in propensity score matching**

| **Matching variable** | **Code set / definition** |
| --- | --- |
| Age at index | TriNetX demographic variable |
| Sex | TriNetX demographic variable |
| Body mass index >30 kg/m² | BMI laboratory/vital record >30 kg/m² |
| White race | TriNetX race variable |
| Black or African American race | TriNetX race variable |
| Asian race | TriNetX race variable |
| Essential hypertension | ICD-10-CM I10 |
| Disorders of lipoprotein metabolism and other lipidemias | ICD-10-CM E78 |
| Overweight and obesity | ICD-10-CM E66 |
| Neoplasms | ICD-10-CM C00–D49 |
| Ischemic heart diseases | ICD-10-CM I20–I25 |
| Nicotine dependence | ICD-10-CM F17 |
| Chronic kidney disease | ICD-10-CM N18 |
| Diseases of liver | ICD-10-CM K70–K77 |
| Type 2 diabetes mellitus with neurological complications | ICD-10-CM E11.4 |
| Type 2 diabetes mellitus with kidney complications | ICD-10-CM E11.2 |
| Cerebrovascular diseases | ICD-10-CM I60–I69 |
| Heart failure | ICD-10-CM I50 |
| Other chronic obstructive pulmonary disease | ICD-10-CM J44 |
| Type 2 diabetes mellitus with ophthalmic complications | ICD-10-CM E11.3 |
| Type 2 diabetes mellitus with circulatory complications | ICD-10-CM E11.5 |
| Noninfective enteritis and colitis | ICD-10-CM K50–K52 |
| Alcohol-related disorders | ICD-10-CM F10 |
| Malnutrition | ICD-10-CM E40–E46 |
| Antilipemic agents | TriNetX medication class CV350 |
| Glucocorticoids | ATC H02AB |
| Metformin | RxNorm 6809 |
| Insulins and analogues | ATC A10A |
| Glucagon-like peptide-1 analogues | ATC A10BJ |
| Sodium-glucose cotransporter-2 inhibitors | ATC A10BK |
| SARS-CoV-2 mRNA vaccine | RxNorm 2468231 |
| Estimated glomerular filtration rate ≥60 mL/min/1.73 m² | TriNetX laboratory term TNX:8001; eGFR ≥60 mL/min/1.73 m² |
| Hemoglobin ≥12 g/dL | TriNetX laboratory term TNX:9014; hemoglobin ≥12 g/dL |
| Albumin ≥3.5 g/dL | TriNetX laboratory term TNX:9045; albumin ≥3.5 g/dL |
| Hemoglobin A1c ≥9% | TriNetX laboratory term TNX:9037; HbA1c ≥9% |
| Thyroid-stimulating hormone within reference range | TriNetX laboratory term TNX:9040; TSH 0.40–4.00 m[IU]/L |
| Thyroid-stimulating hormone test | CPT 84443 |
| Diagnostic ultrasound procedures of the head and neck | TriNetX procedure term 1010760 |
| Ultrasound of soft tissues of head and neck | CPT 76536 |
